# Getting started with Podcasts

**DOI:** 10.1093/eurpub/ckac129.023

**Published:** 2022-10-25

**Authors:** S Buttigieg

**Affiliations:** 1 EUPHA-DH; 2 Digital Health Malta, Msida, Malta

## Abstract

There are a world of opportunities waiting for public health professionals to get started with a regular audio-visual communication channel. The barrier of entry to start creating your very own podcast has been reduced to a bare minimum. This presentation intends to cover the basics to get started with your own podcast and intends to share the lessons from the author's involvement as a co-host of “The Digital Health Voice” podcast.

